# Characterizing Three Azides for Their Potential Use as *C. elegans* Anesthetics

**DOI:** 10.17912/micropub.biology.000794

**Published:** 2023-04-03

**Authors:** Shasha Tu, Jiangyun Li, Kui Zhang, Jianping Chen, Wenxing Yang

**Affiliations:** 1 Department of Physiology, West China School of Basic Medical Sciences & Forensic Medicine, Sichuan University, Chengdu, Sichuan, China; 2 Department of Forensic Pathology, West China School of Basic Medical Sciences & Forensic Medicine, Sichuan University, Chengdu, Sichuan, China; 3 Department of Pathogenic Biology, West China School of Basic Medical Sciences & Forensic Medicine, Sichuan University, Chengdu, Sichuan, China

## Abstract

Sodium azide (NaN _3_ ) is widely used as an anesthetic in the *C. elegans* community for studying animal behavior. It is not known whether other azides can function as anesthetics. This is quite important for the *C. elegans* labs in which NaN _3_ is not a convenient choice, such as all the labs located in China, where NaN _3_ is under tight regulation, and alternative anesthetics need to be characterized. In the present study, we focused on another three azides, potassium azide (KN _3_ ), trimethylsilyl azide (TMSA), and diphenyl phosphoryl azide (DPPA), which are not regulated in China. We characterized their performance in chemotactic behavioral assays and buffer-based assays. Our results suggest that KN _3_ can immobilize worms as effectively as NaN _3_ in the above-mentioned assays. Therefore, we recommend KN _3_ as a routine anesthetic for *C. elegans* labs.

**Figure 1. KN 3 as an alternative anesthetic for C. elegans -related experiments:**
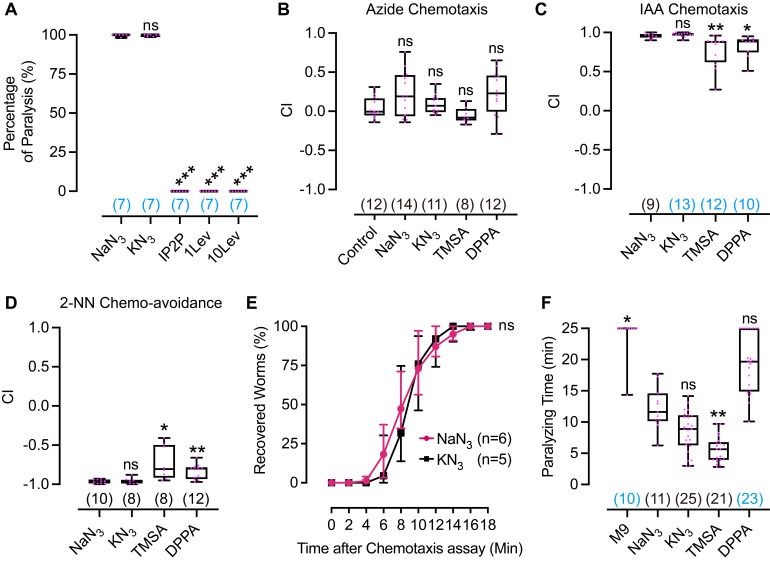
(A) Levamisole (Lev) and 1-Phenoxy-2-propanol (1P2P, 10%) are ineffective at paralyzing worms in chemotaxis assays to IAA. 1Lev, 1 mM levamisole; 10Lev, 10 mM levamisole. (B) All tested azides do not evoke chemotactic behavior. (C) IAA chemotaxis doesn’t change using KN _3_ as an anesthetic, while it changes significantly using either TMSA or DPPA as an anesthetic. (D) 2-NN chemo-avoidance doesn’t change using KN _3_ as an anesthetic, while it changes significantly using either TMSA or DPPA as an anesthetic. (E) Time courses showing the recovery of the worms in 18 minutes after the chemotaxis assays, statistical analysis was performed by comparing the area under the curve. (F) NaN _3_ , KN _3_ , TMSA, and DPPA all showed a certain extent of paralyzing effect in the buffer-based assay. Data in (A)-(D) and (F) are presented as box plots with all data points in magenta; the box plot displays the median, interquartile range, and minimal and maximal values. Data in (E) is presented as a line graph showing the median and interquartile range for the data at the indicated time point. Numbers above the x-axis denote the sample scale of each group; if the number is colored blue, the data is not normally distributed. *vs* NaN _3_ in (A) and (C)-(F); *vs* control in (B); *, *p* < 0.05; **, *p* < 0.01; ***, *p* < 0.001; ns, not significant.

## Description

Sodium azide (NaN _3_ ) is a colorless and odorless chemical widely used as a preservative or anesthetic in scientific community (Chang and Lamm, 2003; Delgado-Cortes et al., 2015; Parida et al., 2014) . NaN _3_ is highly toxic by rapidly depleting intracellular ATP through inhibiting cytochrome oxidase (Tsubaki, 1993) . NaN _3_ treatment induces hypoxia in *C. elegans* , which eventually leads to death (Anyanful et al., 2005) . NaN _3_ can quickly immobilize nematodes, which makes it an ideal tool for paralyzing worms in behavioral tests (Margie et al., 2013) or imaging-related work (Chen et al., 2020; Greene et al., 2016) . Because of its toxicity, NaN _3_ is under tight regulation in some countries, such as China. According to the *Hazardous Chemical Classification Information Sheet* issued by the Chinese government in 2015, sodium azide (Cas No. 26628-22-8), barium azide (Cas No. 18810-58-7), and lead azide (Cas No. 13424-46-9) are under regulation. Thus, keeping NaN _3_ in the lab involves labor-consuming paperwork in China, and finding alternatives may benefit the *C. elegans* community in China.

Except for NaN _3_ , levamisole and 1-Phenoxy-2-propanol (1P2P) have also been used as reagents to immobilize worms in *C. elegans* -based experiments (Manjarrez and Mailler, 2020) . Surprisingly, it seems that almost all published work used NaN _3_ , rather than levamisole or 1P2P or other azides, as an anesthetic to immobilize worms in the chemotaxis assay. To test if levamisole and 1P2P are effective in the chemotaxis assay, we compared their performance with NaN _3_ and potassium azide (KN _3_ ) in the chemotaxis assay. We recorded the worms’ behavior at the end of 1-hour chemotaxis assays with isoamyl alcohol (IAA). When NaN _3_ or KN _3_ is used in the chemotaxis assay, worms were all frozen at the end of the assay ( Fig. 1A ). Neither levamisole nor 1P2P was able to paralyze worms in the chemotaxis assay ( Fig. 1A ), which is quite surprising as it is well known that the worms can be effectively paralyzed when they were soaked in the liquid containing levamisole or 1P2P. These results imply that azide may have a stronger and more acute effect in freezing the movement of the worms, which is quite important for the chemotaxis assay.

To find candidate azides, we searched the online database Chemtown.cn (www.chemtown.cn). We decided to use KN _3_ , trimethylsilyl azide (TMSA), and diphenyl phosphoryl azide (DPPA) in this project based on the following criteria: 1) the chemical is not listed in the *Hazardous Chemical Classification Information Sheet* issued by the Chinese government in 2015; 2) the chemical contains an N _3_ group; 3) the price of the chemical is affordable. An ideal anesthetic for a chemotaxis assay should be odorless to *C. elegans* . We used the chemotaxis assay to test if any of these four azides cause a certain level of chemotactic behavior in *C. elegans* ( Fig. 1B ). As expected, when none of the anesthetics was used, worms spread everywhere on the agar plate, and generated a chemotaxis index (CI) around 0 (“control” in Fig. 1B ), suggesting worms had no preference for either side of the plate. When NaN _3_ , KN _3_ , TMSA, or DPPA was tested, worms were immobilized around the spots where the anesthetic was located, and generated a CI around 0 ( Fig. 1B ), indicating that all four azides are odorless to *C. elegans* . The median CIs in assays using NaN _3_ or DPPA as an anesthetic were about 0.2, implying a potential but weak attraction for worms, although they did not reach statistical significance.

NaN _3_ is a commonly used anesthetic in *C. elegans* research community (Manjarrez and Mailler, 2020) . It is either placed at the supposed destination spot in the behavior assay or dissolved in the buffer or agarose pad (Greene et al., 2016; Parida et al., 2014; Ventimiglia and Bargmann, 2017; Yang et al., 2022) . For the behavior assay, the NaN _3_ can permeate into the agar, which can paralyze the nematodes when the nematodes approach this area. If the anesthetic is not effective, when the worms reach the destination spot, they may move away when they adapted to the odor, which may lead to a change in the CI. Thus, if the anesthetic doesn’t change the CI of worms in the chemotaxis assay, it is likely that the anesthetic is effective. To test the effectiveness of the candidate azides, we challenged the *C. elegans* with an IAA chemotaxis assay (Matsuki et al., 2006; Yang et al., 2022) or 2-nonanone (2-NN) chemo-avoidance assay (Yang et al., 2022) , and used 1 μL of 1 M NaN­ _3_ , or KN _3_ , or TMSA, or DPPA as the anesthetic, respectively. According to our observations, compared to using NaN _3_ as an anesthetic, using KN _3_ as an anesthetic didn’t change the CI in either the IAA chemotaxis assay ( Fig. 1C ) or 2-NN chemo-avoidance assay ( Fig. 1D ). However, when TMSA or DPPA was used as the anesthetic, the absolute CI values were significantly lower in chemotaxis to IAA or 2-NN ( Fig. 1C and D), implying the paralyzing effect of TMSA or DPPA is weaker than that of NaN _3_ or KN _3_ . Our results suggest that KN _3_ , TMSA, and DPPA are all able to paralyze *C. elegans* in agar plate-based behavior assay, while only KN _3_ has a comparable effect with NaN _3_ . When worms were paralyzed by NaN _3_ , they can recover from paralysis gradually. To test if NaN _3_ - or KN _3_ -paralyzed worms can recover after agar-based assays, we collected the worms after the chemotaxis assays and left them on an OP50-seeded NGM plate, then observed their recovery. As shown in Fig. 1E, worms can gradually recover in 18 minutes, and the recovery time courses were similar. These results suggest that NaN _3_ and KN _3_ freeze worms from moving by paralyzing instead of killing them.

A relatively lower concentrated NaN _3_ buffer is widely used as an anesthetic for worm-related imaging (Fang-Yen et al., 2012; Luke et al., 2014; Rajasekharan et al., 2018) . To test if KN _3_ , TMSA, or DPPA can effectively paralyze the worms in such an experimental setting, we soaked the worms in M9 buffer containing 25 mM of different azides. We recorded the worms’ behavior in M9 buffer with or without an anesthetic and compared their paralyzing time, which indicates the time worms take to be immobilized in the buffer. Our results show that worms remained largely motile in M9 buffer within a 25-minute observation window, while NaN _3_ , KN _3_ , TMSA, and DPPA all showed a certain level of paralyzing effect in the M9 buffer, among which TMSA was most effective ( Fig. 1F ).

Based on the above findings, KN _3_ can generate a NaN _3_ -comparable paralyzing effect in both the chemotaxis and the buffer-based assays. Considering NaN _3_ is tightly regulated in China, KN _3_ is especially recommended for research labs located in China. In addition, although TMSA and DPPA did show a certain level of ability to paralyze the worms, we do not suggest them as routine anesthetics in *C. elegans* labs based on the following reasons. Firstly, our data suggest that the paralyzing effect of TMSA and DPPA in the agar-based assay is significantly weaker than that of NaN _3_ and KN _3_ . Secondly, DPPA appeared to have a relatively weaker ability to induce paralysis in worms in the M9 buffer, possibly because it is insoluble in water. Thirdly, we observed that both TMSA and DPPA release irritating odors that can be inhaled by people, suggesting that they can evaporate. Given the potentially toxic effects of azides, it may be unsafe to use TMSA or DPPA in normal lab conditions without taking extra precautions to protect lab members from inhaling the vapors.

## Methods

**Animals**

Wildtype strain (N2) of *C. elegans* was obtained from Caenorhabditis Genetics Center (CGC), which was maintained at 20°C in a 6-cm NGM-agar (nematode growth medium) plate, following previously published protocol (Brenner, 1974) . Synchronized day 1 adult hermaphrodites were used for the assays.

**Chemotaxis Assay and Chemo-avoidance Assay**

The concentrations of the anesthetics used in this manuscript are 1 M for azide, 10% in ethanol for 1-Phenoxy-2-propanol, and 1 or 10 mM for levamisole. Both assays were performed as previously published works (Yang et al., 2022) . Briefly, 1 μL of the anesthetic was placed at two spots, both of which were lying on one line, hereafter referred to as the diameter line, across the center of the 9-cm assay plate and apart from the edge of the agar about 1.5 cm. 100-200 synchronized day 1 adult worms were washed 4 times by M9 buffer (3 g/L KH _2_ PO _4_ , 6 g/L Na _2_ HPO _4_ , 5 g/L NaCl, 0.12 g/L MgSO _4_ ) and placed in the center of the 9-cm assay plate. 1 μL of 100x diluted isoamyl alcohol (IAA) or 1 μL of pure 2-nonanone was placed 2 mm aside from the anesthetic on one side of the assay plate, and 1 μL of M9 buffer or ethanol was placed symmetrically to the odorant on the other side of the assay plate. The residual M9 buffer surrounding the worms was absorbed by Kimwipes™ Delicate Task Wipers. Then the plate was covered by its lid and sealed with parafilm, and the observation immediately started. One hour later, the assay plate was divided into three areas by two lines perpendicular to the diameter line, these two lines were 3 cm apart from each other and the nearest end of the diameter line. N _IAA_ represents the number of worms on the IAA side, N _2-nona_ represents the number of worms on the 2-nonanone side, N _other_ represents the number of the worms on the opposite side to the odor, N _total_ represents the total number of the worms on the plate. The chemotaxis index (CI) is calculated as follows. For the IAA chemotaxis assay, CI = (N _IAA_ - N _other_ )/N _total_ . For 2-nonanone chemo-avoidance assay, CI = (N _2-nona_ - N _other_ )/N _total_ . A CI of nearly 0 suggests no obvious chemotaxis, CI > 0 suggests attraction, and CI < 0 suggests avoidance.

For quantifying the paralyzing effect of NaN _3_ , KN _3_ , TMSA, and DPPA in chemotaxis assay using agar plates, the worms’ behavior was recorded at the end of the 1-hour chemotaxis assay with IAA. The worms that were unable to crawl or twitch were considered immobilized, all other worms were considered motile.

The worms’ chemotactic behavior toward specific anesthetic was observed using the above-mentioned protocol to test if the anesthetic is odorless. For control, no azide was used. For testing NaN _3_ , KN _3_ , TMSA, and DPPA in Fig. 1B, N aN _3_ was used as the anesthetic. CI was calculated as above described.

**Recovery observations**

The chemotaxis assays to IAA were performed using NaN _3_ or KN _3_ as the anesthetics. Immediately after the assay, immobilized worms were picked up and transferred to an OP50-seeded NGM plate. The number of moving worms was counted every two minutes, and the percentage of recovered worms was calculated.

**Paralyzing Assay in M9 Buffer**

NaN _3_ , KN _3_ , and TMSA were dissolved in double distilled water at 1 M, then diluted to 25 mM by M9 buffer. DPPA was dissolved in pure ethanol at 1 M, then diluted to 25 mM by M9 buffer. The video was recorded by a handphone camera fixed on the stereomicroscope using an adapter. For quantification purposes, the time point from which the worm was immobilized for the following 30 seconds was defined as the paralyzing time of the worm being observed.

**Data Analysis**

One-way ANOVA with Dunnett’s multiple comparisons test (if data are normally distributed), Kruskal-Wallis test with Dunn’s multiple comparisons test (if data are not normally distributed), or unpaired *t* test was used to analyze the data. *p* < 0.05 was considered statistically significant.

## Reagents

**Table:** 

STRAIN	GENOTYPE	AVAILABLE FROM
N2	Caenorhabditis elegans	CGC
